# Designing, Prototyping and Evaluating Digital Mindfulness Applications: A Case Study of Mindful Breathing for Stress Reduction

**DOI:** 10.2196/jmir.6955

**Published:** 2017-06-14

**Authors:** Bin Zhu, Anders Hedman, Shuo Feng, Haibo Li, Walter Osika

**Affiliations:** ^1^ School of Computer Science and Communication KTH Royal Institute of Technology Stockholm Sweden; ^2^ School of Film and Animation China Academy of Art Hangzhou China; ^3^ Department of Clinical neuroscience Karolinska Institutet Stockholm Sweden

**Keywords:** respiration, biofeedback, mindfulness, stress, device design, sound, light, breathing, heart rate, relaxation

## Abstract

**Background:**

During the past decade, there has been a rapid increase of interactive apps designed for health and well-being. Yet, little research has been published on developing frameworks for design and evaluation of digital mindfulness facilitating technologies. Moreover, many existing digital mindfulness applications are purely software based. There is room for further exploration and assessment of designs that make more use of physical qualities of artifacts.

**Objective:**

The study aimed to develop and test a new physical digital mindfulness prototype designed for stress reduction.

**Methods:**

In this case study, we designed, developed, and evaluated HU, a physical digital mindfulness prototype designed for stress reduction. In the first phase, we used vapor and light to support mindful breathing and invited 25 participants through snowball sampling to test HU. In the second phase, we added sonification. We deployed a package of probes such as photos, diaries, and cards to collect data from users who explored HU in their homes. Thereafter, we evaluated our installation using both self-assessed stress levels and heart rate (HR) and heart rate variability (HRV) measures in a pilot study, in order to measure stress resilience effects. After the experiment, we performed a semistructured interview to reflect on HU and investigate the design of digital mindfulness apps for stress reduction.

**Results:**

The results of the first phase showed that 22 of 25 participants (88%) claimed vapor and light could be effective ways of promoting mindful breathing. Vapor could potentially support mindful breathing better than light (especially for mindfulness beginners). In addition, a majority of the participants mentioned sound as an alternative medium. In the second phase, we found that participants thought that HU could work well for stress reduction. We compared the effect of silent HU (using light and vapor without sound) and sonified HU on 5 participants. Subjective stress levels were statistically improved with both silent and sonified HU. The mean value of HR using silent HU was significantly lower than resting baseline and sonified HU. The mean value of root mean square of differences (RMSSD) using silent HU was significantly higher than resting baseline. We found that the differences between our objective and subjective assessments were intriguing and prompted us to investigate them further.

**Conclusions:**

Our evaluation of HU indicated that HU could facilitate relaxed breathing and stress reduction. There was a difference in outcome between the physiological measures of stress and the subjective reports of stress, as well as a large intervariability among study participants. Our conclusion is that the use of stress reduction tools should be customized and that the design work of mindfulness technology for stress reduction is a complex process, which requires cooperation of designers, HCI (Human-Computer Interaction) experts and clinicians.

## Introduction

High levels of stress and information overload are increasingly contributing to the global burden of disease [1,2] at great societal costs. For example, in Europe, the total costs of mental health disorders are estimated to be €240 billion per year [3], and globally the costs are most certainly much higher. There is therefore an urgent need for both preventive interventions aimed at reducing the adverse effects of stress, taking into account biological, psychosocial, and environmental risk factors, as well as new effective treatment strategies.

The human stress response is partially mediated through the autonomic nervous system (ANS), divided into the sympathetic and parasympathetic systems. The majority of our internal organs are, directly or indirectly, controlled through these systems. The sympathetic system prepares us for dangerous situations and triggers the stress response. The parasympathetic system governs rest and digestion [4]. High levels of stress and prolonged stress without recuperation result in cognitive, emotional, and somatic symptoms [5-7].

Western stress reduction methods have lately been CBT-oriented (building on insights from Cognitive Behavioral Therapy), with mechanistic approaches, and have emphasized behavioral changes. Eastern meditative traditions have in contrast focused more on present-centered attention and awareness, that is, what has been termed mindfulness in the West [8,9], and non-judgmental acceptance of encountered life situations. Practicing compassion and selflessness in search of balance and resilience, appreciation of life and nature in a fluid and dynamic state of constant imbalance are also major topics of eastern meditative traditions such as Buddhism [10]. Balance and resilience can be achieved through the self-healing capacity of individuals particularly when confronting stress and adversity [11]. Eastern meditative approaches have been deployed for stress reduction in westernized contexts, for example, through the practice of tai-chi [12,13], Qigong [14,15], and mindfulness [16,17].

Drawing on eastern meditative traditions, mindfulness-based stress reduction (MBSR) [18] has been proven effective for reducing stress and anxiety, and is currently one of the most researched treatment packages [19-25]. MBSR is based on procedures to establish increased awareness of moment-to-moment experience and develop compassionate non-judgmental acceptance of oneself, others, and encountered life situations. With digital technologies potentially revolutionizing health and well-being, people increasingly turn to technology to handle stress, anxiety, social isolation, and negative emotions [26-28]. Conventional mindfulness practice is moving to digital devices that potentially can support people’s needs in the digital age [29,30]. New partnerships among psychologists, social scientists, designers, and engineers are needed to better understand the psychological and behavioral impact of these new technologies and applications [31].

In this paper, we focus on designing digital technology for MBSR, in particular, mindful breathing. Mindful breathing can be defined as calm and conscious deep breathing and is a cornerstone of MBSR [32]. We are often unaware of how we breathe and when stressed we breathe in shallow, tensed ways that furthers the stress. “Take a deep breath” is one of the first things we are likely to say to someone deeply anxious. We intuitively know that calm and deep (abdominal) breathing does us good. Can we support such healthy breathing patterns through technology? This is one of the questions we explore in this paper.

Researchers have developed technologies to support relaxation breathing. Wollmann et al developed an android flight game using resonant frequency breathing to increase heart rate variability (HRV) and improve ANS balance [33]. Khut and Muller [34] designed a biofeedback-based multisensory presentation of heart and breathing rates to support self-awareness in more direct ways than traditional quantified-self-monitoring techniques provide. Khut also developed a biofeedback-based stress-relief app called BrightHearts [35,36]. Heart rate and breathing sensors were used to visualize interactive halos and sounds. Colors, diameters, and saturation of the halos vary according to breathing and heart rate. When one’s heart rate decreases, the color changes from red to green and the pitch decreases. Researchers from Stanford University found visual feedback to be effective for supporting healthy deep breathing and that auditory feedback can further subjective feelings of calm [37]. Others found that calming nature sounds were an effective way of reducing stress in an emergency department. Negative affect scores were lower whereas positive affect scores were the same or higher for participants of a nature-sound intervention group [38] on an affect test in comparison with a control group. Vidyarthi et al [39] created a meditative display by connecting respiration to a peaceful soundscape and developed a psychological framework for media immersion. Furthermore, physical and tangible artifacts have been used with digital technologies to capture and represent respiration and bodily interaction. Influenced by somaesthetics [40], Höök and her colleagues translated experiential insights from long-term practice of Feldenkrais exercises into a design prototype called Breathing Light [41], an enclosed space for reflection utilizing body movements. Sensors measure chest movements that control a lamp so the light changes according to breathing patterns. Their somaesthetic design engaged people by increasing their awareness of internal bodily sensations rather than external processes.

**Figure 1:**
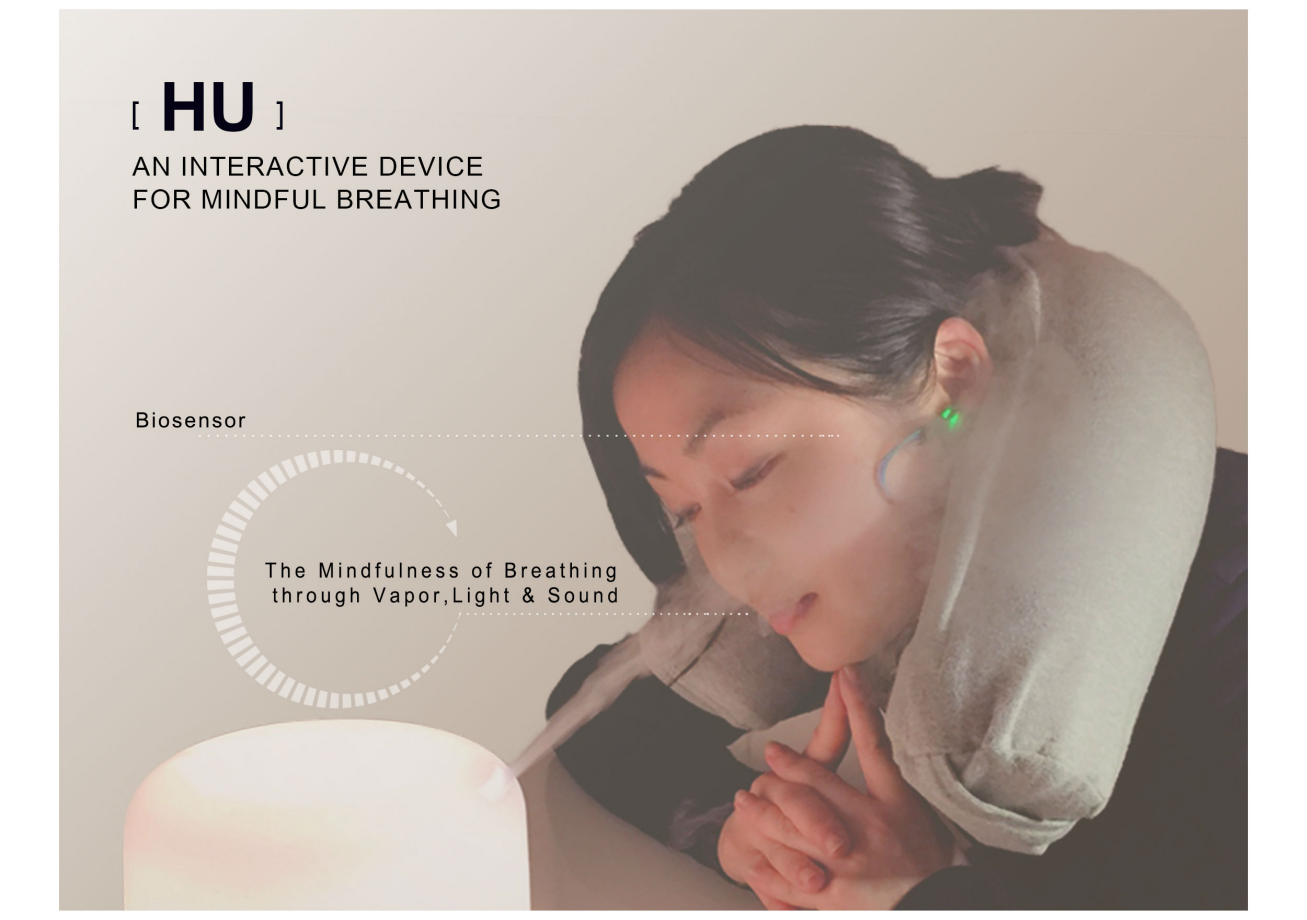
HU: a case study of mindful breathing for stress reduction.

Inspired by the previous studies, we have the hypothesis that interactive physical artifacts based on visual and sound feedback can support mindful breathing for stress reduction. To explore how interactive visual and sound feedback could be used to support mindful and relaxing deep breathing, we designed, developed, and evaluated HU (Figure 1), a physical digital mindfulness prototype based on vapor, light, and sound. HU is a sonified vapor diffuser that expels vapor and emits sounds according to systematic relations with a meditator’s heart rate. We think of these relations as isomorphic and tunable. They are relations among heart rate, vapor expulsions and sounds, subject to experimentation and tuning through user feedback. As a device for supporting mindful breathing, HU could come to function as a part of MBSR interventions.

The paper is structured as follows: First, we look at stress and stress management from scientific and contemplative perspectives, focusing on MBSR. Second, we account for how we developed HU to support mindful breathing for well-being. Third, we describe how users experience HU, as well as how using HU affects them physiologically and psychologically with respect to stress resilience. Finally, we discuss our conclusions and possible directions for future work.

## Methods

### Study Design

We applied the Research through Design (RtD) method [42] in our design process of HU. In an RtD research process, we drive the exploration of both problems and solutions. We gain new knowledge through the act of making things and iterative design work. The knowledge gained during the process can be expressed and articulated in design methods and principles. Our design process consisted of two phases. In the first, initial design phase, we used vapor and light to support mindful breathing. In the second phase, we added sonification.

### Initial Design Phase: The Prototype

The design of HU is inspired by the ancient Chinese incense furnace. In Chinese, HU means to breathe out. HU is a vapor diffuser modified with biosensors. HU measures the heart rate with an earlobe sensor (Figure 1) and supports resonant frequency breathing through vapor pulses. Resonant frequency breathing is a specific biofeedback training strategy based on resonance properties of the cardiovascular system [43,44]. Resonant frequency breathing can be effective in furthering relaxation and homeostatic balance [43]. HU expels vapor to support resonant frequency at 0.1 Hz (6 breaths/minute) [43,45]. The light of HU is adapted with the meditator’s heart rate through the brightness. Fast heart rates are bright and slow heart rates dim.

**Figure 2:**
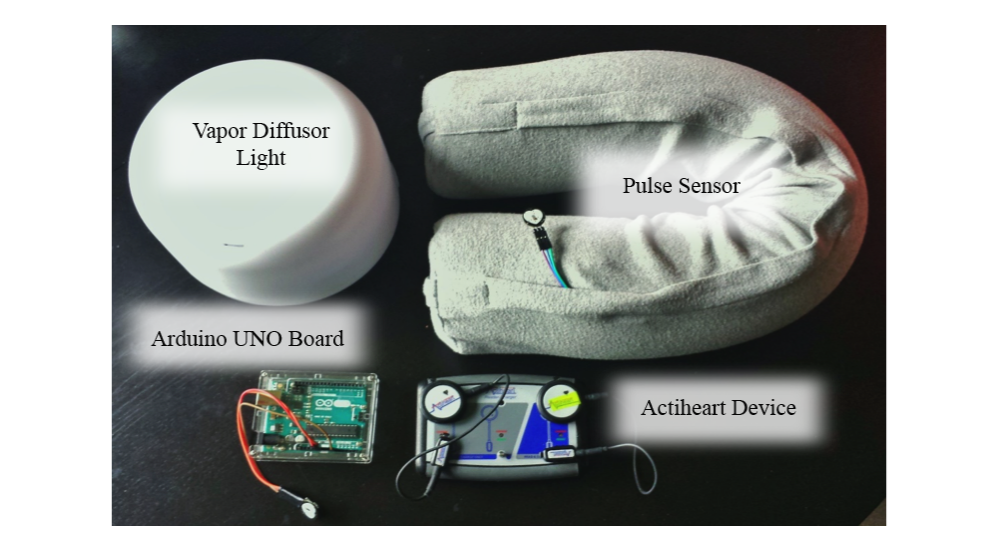
HU prototype.

### Initial Design Phase: Evaluation

We investigated how HU could support mindful deep breathing through a meditation intervention. We selected 25 participants through snowball sampling [46], of which 12 were males and 13 were females. The ages ranged between 23 and 60. They were originally from Sweden (10), China (7), Spain (2), the United States (2), the United Kingdom (1), Italy (1), and Australia (1), and all lived in Sweden. They represented a range of occupations including teachers, students, engineers, doctors, office workers, and mindfulness coaches. Among the 25 participants, 15 lacked the experience of mindfulness or meditative practice; 5 were beginners; and the remaining 5 were experienced mindfulness meditators. We arranged the HU in a quiet room and asked participants to try it for 10-20 min, and then interviewed them. The participants were interested in HU and enjoyed engaging with HU to support mindful breathing. About 22 of the 25 participants (88%) claimed that vapor can lead to mindful breathing and helped them relax. The meditators regarded the vapor as a good component for 4 reasons:

1. Rhythmically tuned vapor pulses can support synchronized deep breathing.

2. Vapor is aesthetically pleasing.

3. Fleeting vapor reflects the fleeting present.

4. Vapor is subtle and non-distractive.

Three meditators, however, thought vapor failed to support mindful breathing. Two of them lacked experience with mindfulness and claimed it was difficult to follow the vapor while focusing on their breath. One of them was a mindfulness coach who had practiced mindfulness for 10 years. She focused better on her “inner being” without the vapor.

Fifteen participants saw the light as a good way to enhance the experience of interaction. Light was regarded as a good medium mainly for three reasons:

1. It is visually pleasing and evokes enjoyment.

2. It enriches the breathing practice.

3. It supports body awareness.

Five participants disliked the light. They felt the connection between the light and heart rate was ambiguous and unclear. Three of them felt the light (in particular, the brightness indicating high heart rate) was stressful. Two mentioned that the rapidly changing light distracted them.

The results from the trial suggested that vapor and light can be effective ways of promoting mindful breathing, but that vapor could potentially support mindful breathing better than light (especially for mindfulness beginners). Finally, we found that, interestingly, a majority of the participants mentioned sound as an alternative medium for mindful breathing. Further to this, 13 participants closed their eyes during the trial, which indicated why sound might seem so natural.

### Sonified Design Phase: The Prototype

According to our meditation intervention feedback, we supplemented HU with sound. Sound can reduce stress [47,48]. Humans normally respond with affect to rhythmical sound [49], and rhythmical sound can in turn influence human behavior [50,51]. Sound can have positive psychological and physiological effects on illness [52-56]. Modern nursing pioneer Florence Nightingale noted that wind instruments, the human voice, and stringed instruments helped patients recover [57]. Alvarsson et al investigated the way sounds of nature affected subjects physiologically and psychologically [58]. Forty trial subjects were exposed to a “sounds of nature” or a “noisy” environment after performing stressful mental arithmetic. The results demonstrated that people recovered faster from sympathetic activation in the “sounds of nature” environment. Another study suggested that a natural environment had restorative effects on patients, including more positive emotions [59] and less mental fatigue [60].

We selected suitable soundtracks from a natural environmental sound database and a music database for our prototype. The prototype has two parts: a hardware prototype and a software application. The core of our hardware prototype (the sense and control unit) includes an Arduino UNO board and a pulse sensor. Heart rate data can be collected via the pulse sensor and transmitted to the Arduino UNO board. Arduino is an open source integrated programming environment [61]. The software is an audio application running on a PC developed using Pure Data, a real-time, graphical, dataflow programming application [62]. The software prototype enabled sound to be coupled with the meditator’s heart rate, so tempo and sound volume increased and decreased synchronously with heart rate. We offered three sound themes:

1. Breathing: A female breathing sound

2. Nature: Rhythmical ocean waves

3. Music: Half Moon Serenade (meditation music)

People could choose sound themes freely.

### Sonified Design Phase: Evaluation

We used two approaches to evaluate HU: an exploration study and an experiment. Here, we name HU without sound as silent HU to distinguish it from sonified HU.

#### Exploration Study

**Figure3:**
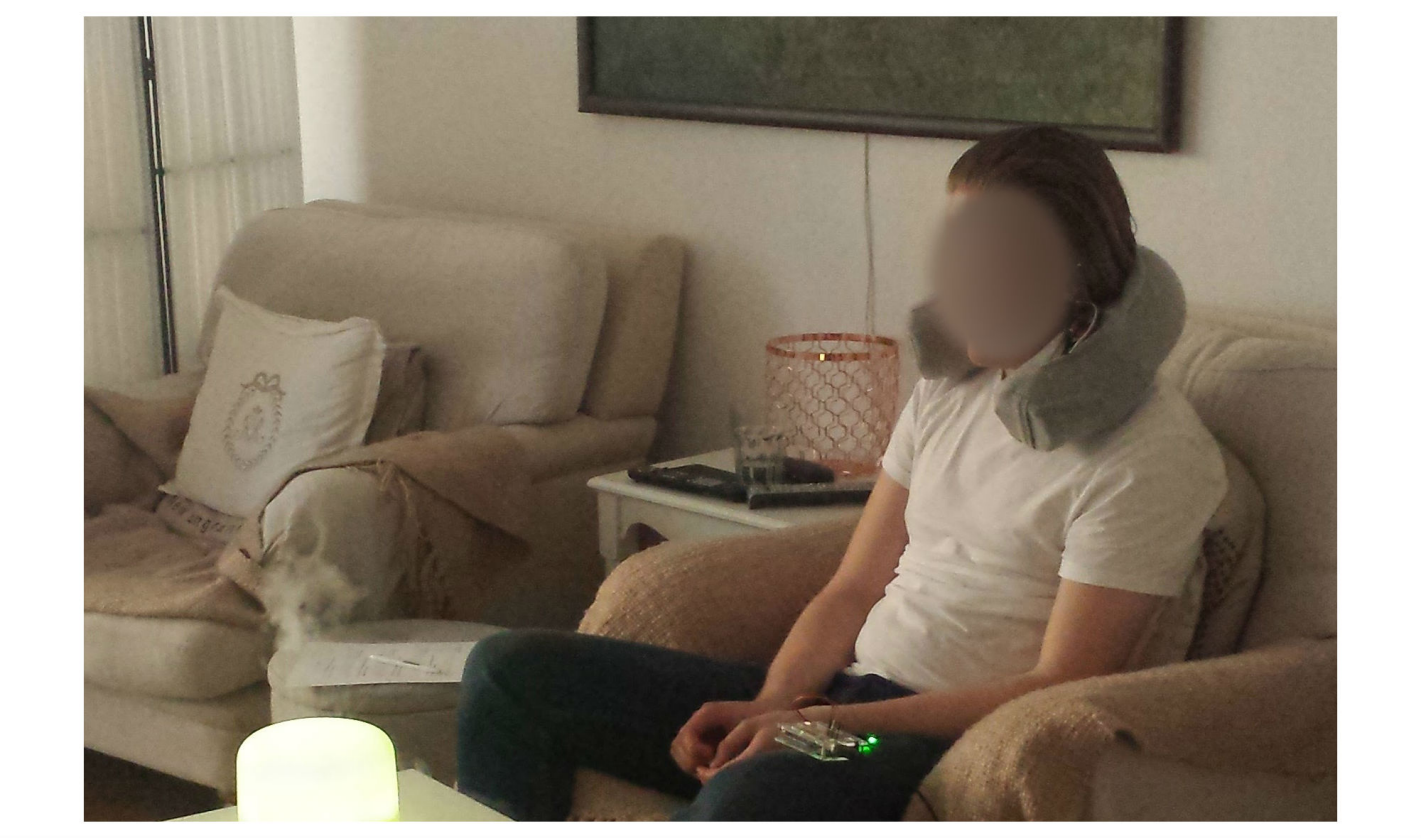
Trial photos in the context.

**Figure 4:**
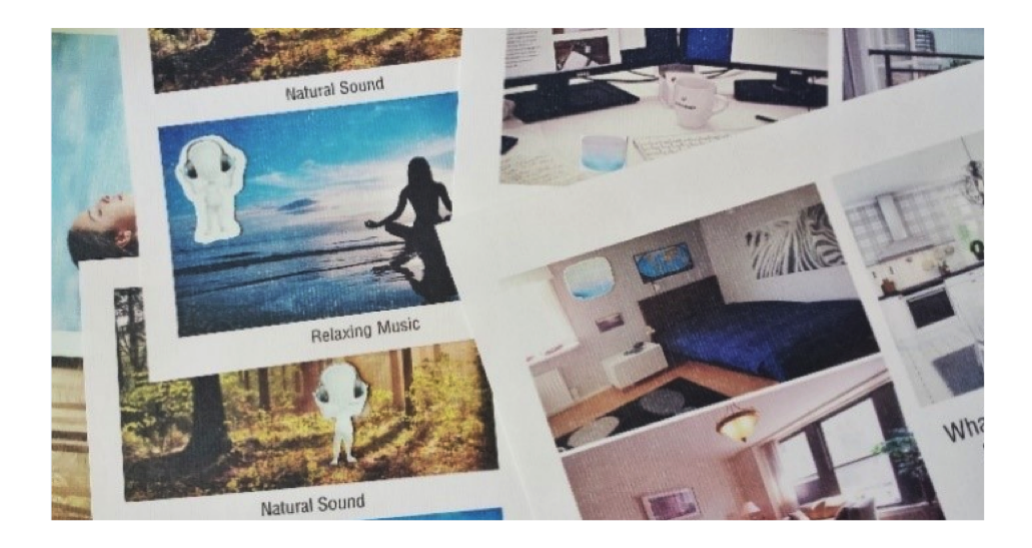
Picture cards.

To study an everyday-use scenario, the experiment was conducted in the participant’s homes (Figure 3). In the exploration stage, participants used HU for 2 days, in accordance with their own likings. During this stage, they described their experiences through diaries, photos, and short videos (of how and where they used HU). The diaries captured use conditions, including time, place, and duration, as well as experiences before and after use. To understand sound preferences for relaxation, we used picture cards on which participants attached premade cartoons to indicate their choices (Figure 4).

**Figure 5:**
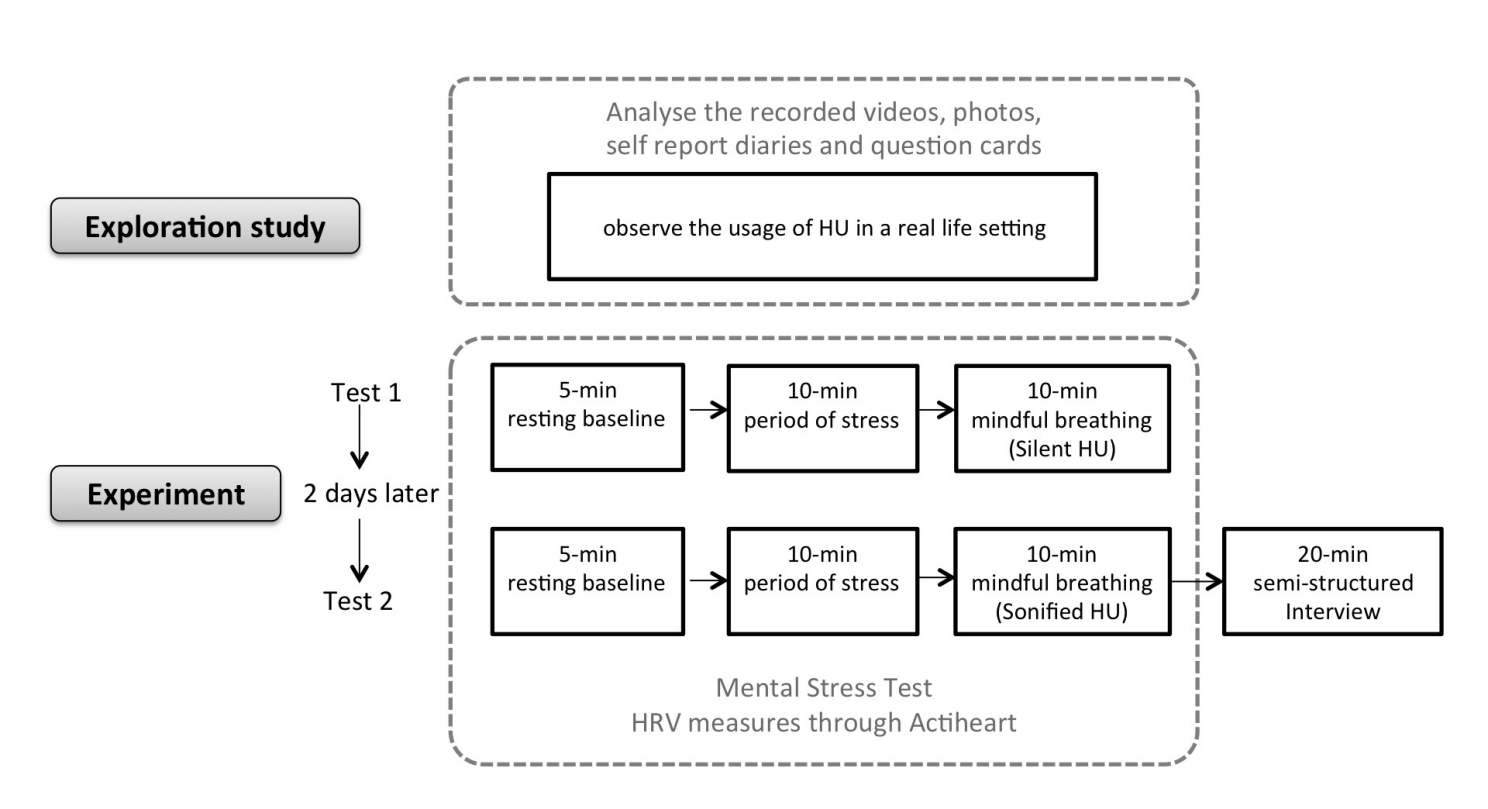
The procedure of the test.

#### Experiment

We conducted an experiment to discern how HU could help reduce stress through mindful breathing and evaluated the outcome through self- assessment and HRV measures.

#### Measures

##### Mental Stress Test

A mental stress test was used as a stress provocation, building on earlier research with mental stress experiments [63]. We used a 10-min mental arithmetic test from the Wechsler Adult Intelligence Scale (WAIS) [64], as for example, the Trier social stress test [65] did not work in our setting. Some research has been performed on the stress inducing effects of WAIS [66-68].

##### Visual Analogue Scale (VAS) for Subjective Stress Assessment

Visual analogue scales (VAS) are often used for assessing subjective stress levels [69-71]. Our participants marked how stressed they felt on a 10 cm long line, where zero distance from the left=No stress, 5 cm=Neutral, and 10 cm=As bad as it could be.

##### HRV Measurements

HRV is the variability of intervals between consecutive heartbeats. It is regarded as a performance indicator of autonomic heart rate regulation and ANS balance [72]. High HRV is associated with a well-balanced ANS, whereas low HRV is associated with chronic stress and diseases such as diabetes and depression [73-75]. HRV has also been used as a measure of mental stress reactivity over short time periods. Troubat et al found that mental stress during short periods was associated with decreased mean HRV [76]. Researchers have sought methods for restoring normal HRV to achieve balance in the ANS [77].

HRV measures are usually divided into two categories: time domain measures and frequency domain measures. One commonly used time-domain measure root mean square of differences (RMSSD) between successive rhythm-to-rhythm (RR) intervals describes short-term HRV. In a study among employees of an electronics company, researchers found that RMSSD value is associated with perceived mental stress [78]. A low value indicates high stress.

Frequency domain method takes into account different frequency components of HRV. The two main frequency components that represent ANS activity are the low-frequency (LF) components (0.04-0.15Hz) and the high frequency (HF) components (0.15-0.4 Hz). The LF band reflects sympathetic activity and the HF band, the parasympathetic activity. The ratio of LF to HF power (LF:HF) has been used to calculate and assess the sympathovagal balance for indicating the function of the ANS activity [79,80].

The Actiheart device (CamNtech, UK) [81] was used for data sampling and primary data analysis in our study. It is a compact, chest-worn monitoring device that records heart rate, interbeat interval (IBI), and physical activity in one combined, lightweight waterproof unit. In the time domain analysis of HRV, the information of inter-beat interval in each measurement period was computed. In the frequency domain analysis, spectral analysis was performed.

#### Recruitment

Five new participants were recruited through word of mouth (3 males and 2 females, mean age 23 years). All participants were from universities in Sweden, with normal vision and hearing. Before the test, we gathered basic demographic information and their individual assessments of stress levels for the last week. The participants signed consent forms and allowed us to use their data. We used a within-subject design to evaluate silent HU and sonified HU, respectively. As perceived stress and HRV varied from individual to individual, a within-subject design was selected to reduce error variance associated with individual differences [82].

#### Procedure

Figure 5 shows the test procedure schematically. The experiment comprised 3 sessions:

1. 5-min resting baseline: Participants wore Actiheart and sat in a comfortable position as their resting baseline HRV was sampled.

2. 10-min period of stress: In order to observe the effect of using HU for stress reduction, we triggered a stress response in participants before the mindful breathing task. The stress-generating task was the 10-min IQ test from the Wechsler Adult Intelligence Scale (WAIS), described above.

3. 10-min mindful breathing with HU: HU was used to guide mindful breathing for 10 min. In a first test, participants used silent HU in the mindful breathing period. After 2 days, they used sonified HU in a second test.

During the tests, participants sat in an undisturbed environment. Heart rate, RMSSD, HF, and LF were measured through the Actiheart device (Figure 2). The visual analog scale described earlier was used for assessing subjective stress levels after each session of the test.

After the experiment, we performed a semistructured interview with questions organized in 3 sections. The first section contained questions about the participants’ ways of relieving stress in everyday life. The second section contained questions about HU that did not have to do with the sonification (eg, how they had used HU in their everyday life and whether they could relate the vapor flow or light to their heart rate). The last section explored issues related to the sonification. First, they were asked to describe their experiences with the sonification, and that whether they could relate the sound rhythms to their heart rate. Subsequently, we asked them what characters of sound (pitch, tempo, and volume) would make them comfortable, and how the sonification could be improved.

## Results

### Sonified Design Phase: Results From the Exploration Study

In the exploration stage, we observed the usage of HU in home settings with 5 participants as described earlier and analyzed their recorded videos, photos, and self-report diaries. The 5 participants reported different degrees of relaxation during and after engaging with HU. Three reported that HU guided them to breathe mindfully.

When I used it, I know it worked with my breath and enabled me to follow it and take away my focus from the work. It makes me calm and peaceful…

P1

I felt relaxed when I breathed with HU. After that, I studied more efficiently…

P4

Three of them used HU in the evening and before going to bed. One preferred the morning and another chose the afternoon time for relaxation. They used HU for 10-20 min at a time. Most of them installed HU in their bedrooms and living rooms for mindfulness and relaxation. One used HU on a desk beside her while working. The bedroom was the most popular location for HU.

Apart from engaging with HU to support mindful breathing, they explored how HU, as an artifact, could fit into their daily life. To illustrate this last point, some found that HU could serve as a humidifier or ambient light, with the added benefit of enhancing calmness and relaxation.

It just makes me feel good when HU is besides me.

P2

I like the dim yellow light and the vapor. Especially the white vapor…looks dreamy…

P3

I used HU as a humidifier at home. It works for me…I like a multi-functional device...could be a breath machine for meditation, a humidifier or an ambient light…

P5

In terms of the 3 interactive sound themes, 2 out of 5 participants thought nature sounds best helped them relax. They commented that the sound of water such as ocean waves could help them relax. The others believed meditation music was more relaxing. The woman’s breathing sound can be used for guiding the breath but was not considered as relaxing as the other two themes.

### Sonified Design Phase: Results From the Experiment

A paired *t* test was used to evaluate the effects of using silent HU and sonified HU among the 5 participants. The HR, RMSSD, LF:HF, and subjective stress scores are shown in Table 1. The mean value of HR using silent HU is significantly lower than the resting baseline and sonified HU (63.4 vs 68.6, *P*=.008; 63.4 vs 68.4, *P*=.03, respectively). The mean value of RMSSD using silent HU is significantly higher than the resting baseline (58.734 vs 46.396, *P*=.04). However, the mean values of RMSSD and LF:HF during use of sonified HU showed no significant improvement compared with silent HU and the resting baseline. The subjective stress assessment was significantly improved in silent HU (3.1 vs 6.54, *P*=.01) and sonified HU (2.9 vs 6.54, *P*=.01) compared with the resting baseline. In terms of subjective stress, there was no significant difference between silent and sonified HU (*P*=.82).

**Table 1:** Mean and *t* test scores during resting baseline and silent HU

Measures	Baseline		Silent HU		*t* test	*P* value
	Mean	Standard error	Mean	Standard error	*t*_4_ degrees of freedom=4	*P* significance level of .05
HR	68.6	2.32	63.4	2.77	4.8702	.008
RMSSD	46.396	8.28	58.734	8.98	−2.9317	.04
LF:HF	2.37	0.89	3.264	1.63	−0.5512	.61
Subjective stress (0-10)	6.54	0.70	3.1	0.56	4.4278	.01

**Table 2:** Mean and *t* test scores during resting baseline and sonified HU

Measures	Baseline		Sonified HU			*t* test		*P* value	
	Mean	Standard error		Mean	Standard error		*t*_4_ degrees of freedom=4		*P* significance level of .05
HR	68.6	2.32		68.4	3.93		0.0960		.93
RMSSD	46.396	8.28		45.094	2.42		−0.1783		.87
LF:HF	2.37	0.89		5.126	2.04		−1.3160		.26
Subjective Stress (0-10)	6.54	0.70		2.9	0.62		4.3993		.01

**Table 3:** Mean and *t* test scores during silent HU and sonified HU

Measures	Silent HU		Sonified HU			*t* test		*P* value	
	Mean	Standard error		Mean	Standard error		*t*_4_ degrees of freedom=4		*P* significance level of .05
HR	63.4	2.77		68.4	3.93		−3.2969		.03
RMSSD	58.734	8.98		45.094	2.42		−1.5197		.2
LF:HF	3.264	1.63		5.126	2.04		−2.3225		.08
Subjective Stress (0-10)	3.1	0.56		2.9	0.62		0.2453		.82

We compared the measures of HR, RMSSD, LF:HF, and subjective stress during each session and analyzed the results individually (in Multimedia Appendix 1).

### Sonified Design Phase: Interview Findings

P1 and P4 thought silent HU worked better than sonified HU. They declared that the visual aids were more effective than the auditory for relieving stress, as the vapor seemed more connected to the breath.

I think it can help me some…it was a relaxing device in that sense. I would observe the light changing for a little while and then close my eyes to relax.

P1

I found the sound and vapor did not correspond to each other. I focused on the vapor more than the sound so that I did not notice the relationship between sounds and my bodily state.

P4

Three more participants were also unsure about the sound and its relation to their bodies. They guessed there might be connections between the sound and their breathing or heart rate, because the music changed when they realigned their postures:

Though sometimes it was a very weird pitch of the sound, I could partly find that the sound corresponded to my breathing when I took a deep breath.

P3

I suspect the pitch and frequency of the audio were affected by my pulse, because when my pulse was high and fast, the sound would be more rapid and at a higher pitch.

P5

I did not know how it worked, but I sometimes found the sound to be a little bit weird and distorted.

P2

Thus, the perceived clarity of mappings between body and visualizations and sonifications should be taken into account in designing interactive interfaces.

We asked concrete questions about the perceived effectiveness of the sonification for stress reduction. Three participants thought sound helped them ease tension and relax. One thought the interactive sound should be changed more gradually. One of them wanted to equip HU with surround sound for an immersive experience. One felt natural sounds such as wave sounds naturally helped that person better than music because of their rhythmic constancy. All thought the music should be simple, so they could focus on their breath and not on the music. Two participants found the changing tempo annoying. One of them put it this way:

Changing the tempo of the music continuously makes it weird. The music becomes distorted, and you focus on the sound. The pitch was going up and down a lot, it is better to have steady music to relax.

P2

One participant was annoyed with the audio clips being repeated. When asked about the dynamic volume, most said they had not noticed the volume changed with their heart rate measures.

## Discussion

### Principal Findings

By developing and testing HU, we found that its vapor, light, and sound could enhance stress reduction. There was much interindividual variability in terms of preferred settings and modes and perceived stress reduction, and HR and HRV measures. Subjective stress levels were significantly improved with both silent and sonified HU. The mean value of HR using silent HU was significantly lower than the resting baseline and sonified HU. The mean value of RMSSD using silent HU was significantly higher than the resting baseline. However, the mean values of RMSSD and LF and HF during the use of sonified HU showed no significant improvement compared with silent HU and the resting baseline. In terms of subjective stress, we found no significant difference between silent and sonified HU.

How the participants perceived the mappings between heart rate and visualizations and sonifications influenced their overall experience. Regarding our interactive sound program that adjusts speed and volume according to the heart rate, there are challenges that need to be addressed. The sound tempo was not the only characteristic affected by heart rate; the pitch too was changed as a side effect. From our observations and interviews, it became clear that such pitch distortions bothered the participants. It is easy to see why: tempo and pitch play effectual and esthetic roles in music and it can be difficult to change both tempo and pitch at the same time while preserving those roles. One possible solution is to keep a slow tempo all the time. We did not test with such a noninteractive sonification, but it could be interesting to compare with in the future. Another possible solution is to use advanced sound processing, so that the pitch is preserved with tempo.

Although the sound volume varied during the experiment, participants did not notice. When we asked them about how changing the volume dynamically might enhance relaxation, they all stated that such volume changes would not affect them. It is unclear to us why sound volume did not seem to matter.

An interesting finding is that P2 and P3 felt sleepy when using HU. Participants felt calm after they used sonified HU, and most of them used HU in their bedrooms. One participant said:

It makes me peaceful and sleepy. Therefore, I think it will be an excellent tool for sleep.

P2

HU could perhaps function as a sleep aid. HU was designed as digital mindfulness artifact, and mindfulness is commonly associated with alert wakefulness. However, most meditative traditions have incorporated measures for keeping mediators awake. It is common, for example, in many schools of Buddhism to hit meditators with a stick on their backs when they fall asleep. Zen masters often do this. As our users engaged with HU, participants who felt sleepy were clearly able to calm down as in mindful mediation and like many other meditators this brought them closer to the sleeping state. A similar finding was reported by Britton et al, that is, that early phases of meditation may produce more fatigue and sleep propensity whereas later stages produce greater wakefulness as a result of neuroplastic changes and more efficient processing [83]. Sleepiness may not be the state that is most desirable in mindful mediation, but it seems to be a phase novice practitioners often go through. This would be interesting to explore in detail in a future study.

Another point to be discussed is the discrepancy between Actiheart measurements and subjective experience. For example, P3 reported feeling more relaxed in the period of sonified HU with a score of 0.5 compared with 3 of silent HU. However, RMSSD and LF and HF measures indicated that sonified HU was associated with more stress than silent HU and the resting period. The post-study interview may help us understand the reason for the discrepancy between subjective and physiological measures. As P3 said:

I do not like that the tempo of the sound changed. If your heart rate is below your initial point, you are very relaxed. However, if your heart rate is higher than a particular point, say 79, the music you used was at very high pace, and you couldn’t feel relaxed...

P3

There was also a discrepancy between physiological measurements and self-reported stress levels in the case of P5. P5 expressed her critical opinion on the dynamic sound:

I do not like this form of the sound. It is acceptable that the tempo is changing but not for the pitch. I also think the rhythm should not be modified a lot.

P5

What is the right way to think about the physiological HRV measures and the self-reported stress measures? We cannot make sense of these measures without taking into account several complicated factors. One factor is for example that of the psychological situation of the test subject; it could be that the test subject is reporting feelings of relaxation while the subject is in fact not experiencing such feelings. The subject might be trying to please the experiment leader. Another factor could be sampling: perhaps the HRV equipment did not sample the signals adequately. A third factor is that the physiological and psychological measures actually might come apart, that is, they do not correspond perfectly. We hope to explore further about the relations between HRV measures and self-reported stress measures in a future follow-up study. We learned from our case study how important it is to work in a cross-disciplinary team and to make sense of the data from different perspectives. Calvo et al have stressed the significance of partnership between psychology, social sciences, and technologists [31] in the domain of human-computer–interaction for well-being. We use objective and subjective (qualitative and quantitative) approaches in terms of the assessment of mindfulness-based stress reduction. HRV measurements are commonly used in stress and medical research. These objective measures of stress are seldom used by researchers in human-computer–interaction and user experience design. The results of our study suggest we should not abandon subjective reports of user experiences. Both subjective and objective methods give valuable information. We found that the differences between the objective and subjective assessments were intriguing and prompt us to investigate them further.

### Limitations

The main limitation of this study was the low number of participants. Moreover, the participants were recruited by a snowball sampling technique. To prove that sonified HU is effective in comparison with silent HU in improving HRV, more users would have to be recruited and according to a random sampling scheme. The participants’ home environments were chosen as the experimental settings, but naturally, it is impossible to control for potentially disturbing factors in the same way that one can do in a lab or dedicated test environment.

### Future Work

This study provoked greater thinking about how to design sonified devices for relaxation and mindfulness. The question of how biodata-based interactive sound could potentially reduce stress, along with the question of whether interactive sound or regular music or sounds has a more positive influence on stress reduction are topics that warrant further exploration. We plan to continue our investigations and design headphones with a pulse sensor and sound files embedded inside. A further challenge with such a device would be to incorporate HRV functionally.

### Conclusions

The primary purpose of this study was to investigate whether a newly developed tool, HU, could support mindful breathing and help users reduce stress after exposure to a mental stressor. The observations, interviews, and HRV data helped us to, if not fully answer this question, explore it. Our study indicates the potential of interaction design based on vapor, light, and sound to support mindful breathing and stress reduction. Further to this, feedback from user explorations of HU in their homes and the interviews helped us understand more about the subjective experiences of using this kind of installation.

## Appendix

Multimedia Appendix 1 Comparison of the measures of HR, RMSSD, LF/HF, and subjective stress during each session and individual analysis of the results.

## Abbreviations

ANS: autonomic nervous system
MBSR: mindfulness-based stress reduction
HR: heart rate
HRV: heart rate variability
