# Supplementary material for: Designing, Prototyping and Evaluating Digital Mindfulness Applications: A Case Study of Mindful Breathing for Stress Reduction
Source: J Med Internet Res. 2017 Jun 14;19(6):e197. doi: 10.2196/jmir.6955 (PMC5489711; doi:10.2196/jmir.6955)
Supplement: Multimedia Appendix 1 [file jmir_v19i6e197_app1.pdf]

Multimedia Appendix

To better understand the subjective evaluations of silent and sonified HU, we analyzed the data individually.

Subjective stress: P1 and P5 reported lower subjective stress levels with silent HU,while P2 and P3 reported lower subjective stress with sonified HU. P4 felt the same subjective stress with silent and sonified HU.

The measures of HR, RMSSD, LF/HF: (As mentioned earlier: lowered RMSSD measures and/or elevated LF/HF scores indicate higher stress levels). P1 exhibited lower HR, higher RMSSD and a decreased LF/HF ratio with both silent and sonified HU compared to resting baseline. Both silent and sonified HU reduced his stress. Through comparing changes between silent HU and sonified HU, we found that silent HU was most supportive for relieving stress and improving ANS performance in P1. The HRV measurement was also consonant with P1’s reported subjective stress.

From the subjective stress questionnaire, it was obvious that P2 felt less stressed when using silent HU (score from baseline 6.5 to 5) and even less stressed when using sonified HU (score 3.5). These findings were also supported by the RMSSD measures. However, the HR and LF/HF ratio indicated that silent HU worked better for stress reduction and restoring normal ANS function.

Regarding subjective stress, P3 felt more relaxed in the period of sonified HU with a score of 0.5 compared with 3 for silent HU. But RMSSD and LF/HF showed that sonified HU induced more stress than sonified HU and the resting baseline. The physiological measurements were thus not in accordance with the subjective assessments.

For P4, all measures except LF/HF showed stress reduction and ANS improvement in the period of silent HU and sonified HU. P4 perceived the same level of stress 3.5 when using silent and sonified HU. But the HR and RMSSD indicated that P4 was in a state with less stress in the period of silent HU. LF/HF increased substantially in the period of silent HU and sonified HU. It also happened in the case of P3 and P5.

P5 reported a significant reduction of stress in the period of silent HU. The HR and RMSSD can support P5’s experience. The LF/HF measure does not.

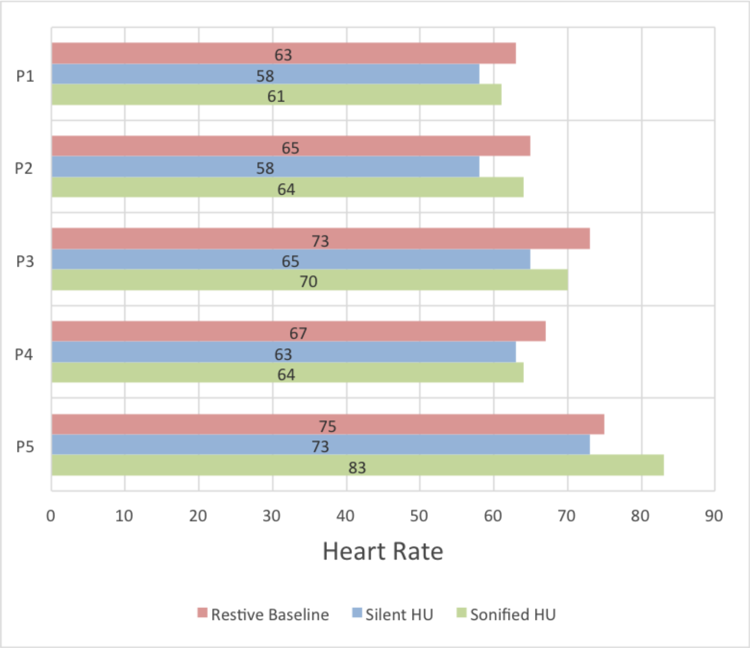

Figure 7. Heart rate measures. Mental stress is associated with increased heart rate

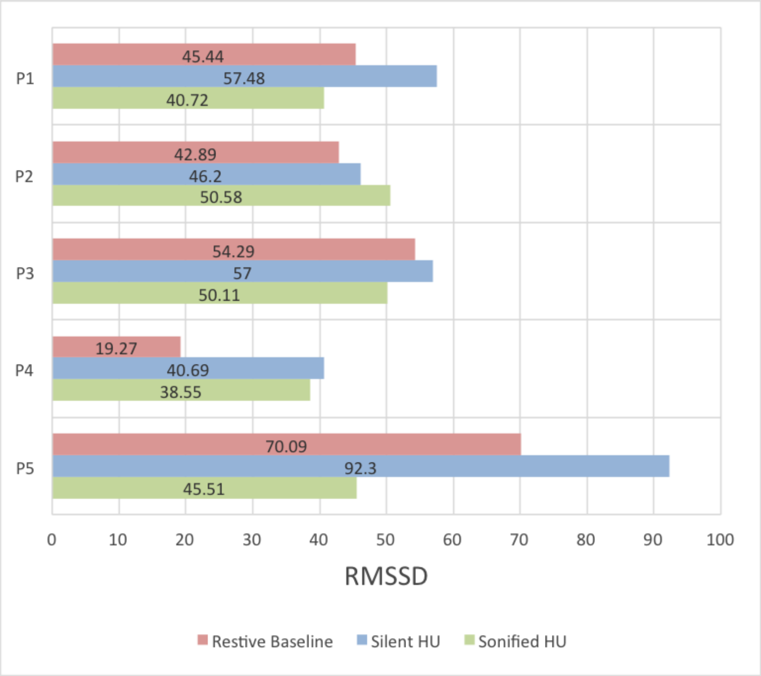

Figure 8. RMSSD measures. Mental stress is associated with decreased RMSSD

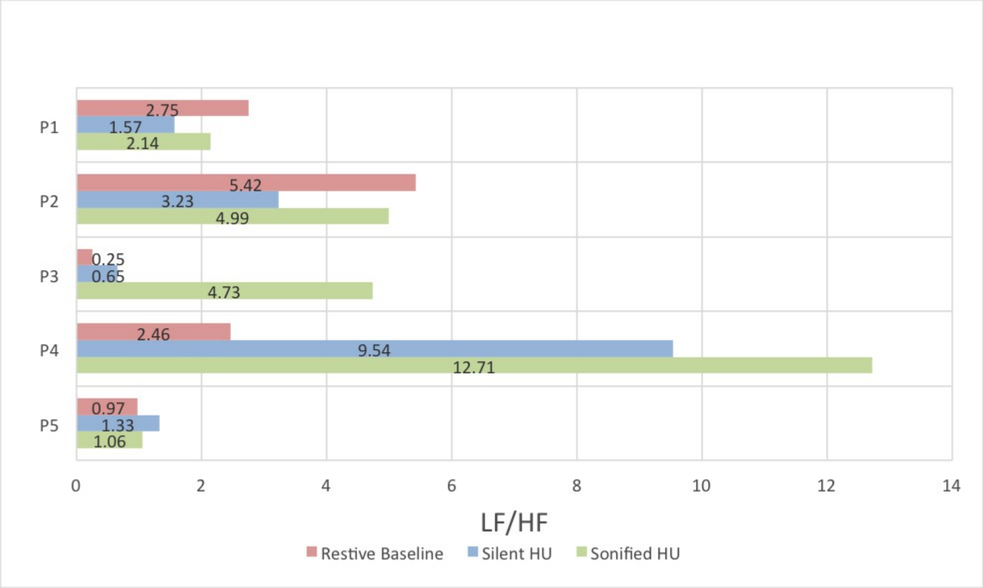

Figure 9. LF/HF measures. Mental stress is associated with increased LF/HF ratio

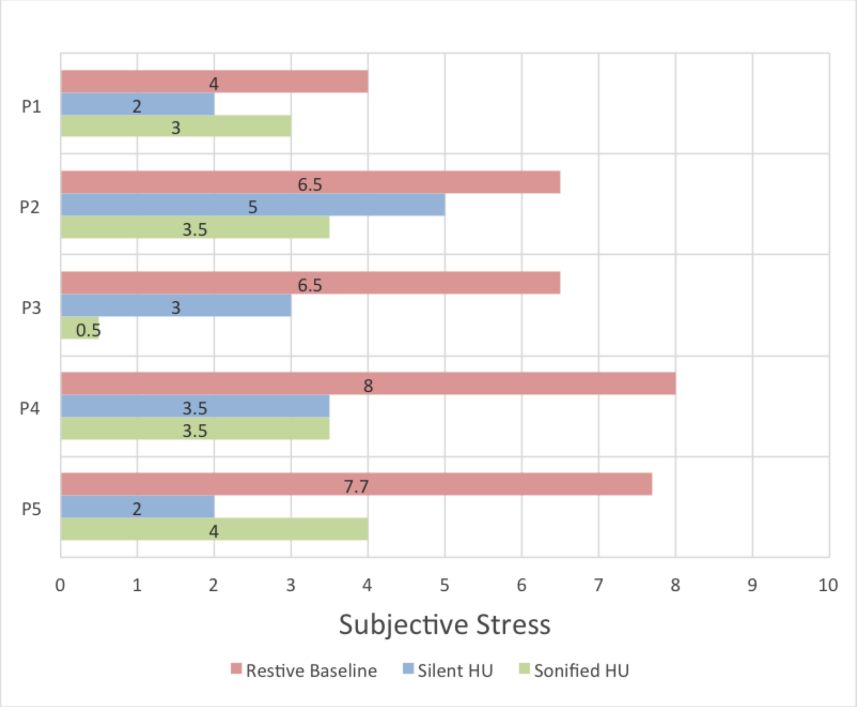

Figure 10. Subjective stress measures
